# Flexible polyimide-based hybrid opto-electric neural interface with 16 channels of micro-LEDs and electrodes

**DOI:** 10.1038/s41378-018-0027-0

**Published:** 2018-10-08

**Authors:** Bowen Ji, Zhejun Guo, Minghao Wang, Bin Yang, Xiaolin Wang, Wen Li, Jingquan Liu

**Affiliations:** 1 National Key Laboratory of Science and Technology on Micro/Nano Fabrication, Department of Micro/Nano Electronics, Shanghai Jiao Tong University, Shanghai, 200240 China; 2 Electrical and Computer Engineering Department, Michigan State University, East Lansing, MI 48824 USA

## Abstract

In this paper, a polyimide-based flexible device that integrates 16 micro-LEDs and 16 IrO_*x*_-modified microelectrodes for synchronous photostimulation and neural signal recording is presented. The 4 × 4 micro-LEDs (dimensions of 220 × 270 × 50 μm^3^, 700 μm pitch) are fixed in the SU-8 fence structure on a polyimide substrate and connected to the leads via a wire-bonding method. The recording electrodes share a similar fabrication process on the polyimide with 16 microelectrode sites (200 μm in diameter and 700 μm in pitch) modified by iridium oxide (IrO_*x*_). These two subparts can be aligned with alignment holes and glued back-to-back by epoxy, which ensures that the light from the LEDs passes through the corresponding holes that are evenly distributed around the recording sites. The long-term electrical and optical stabilities of the device are verified using a soaking test for 3 months, and the thermal property is specifically studied with different duty cycles, voltages, and frequencies. Additionally, the electrochemical results prove the reliability of the IrO_*x*_-modified microelectrodes after repeated pressing or friction. To evaluate the tradeoff between flexibility and strength, two microelectrode arrays with thicknesses of 5 and 10 μm are evaluated through simulation and experiment. The proposed device can be a useful mapping optogenetics tool for neuroscience studies in small (rats and mice) and large animal subjects and ultimately in nonhuman primates.

## Precise multisite control of neural activity with opto-electric device

A durable, flexible device can be implanted on rat brains to precisely turn on nerve cells using light and synchronously record their activities. Jingquan Liu and colleagues from Shanghai Jiao Tong University in China combined reliable wire-bonding micro-LED and modified microelectrode arrays to design a device that can more precisely target local brain cortex with light than the currently used optic fibers in optogenetics. In this field of research, scientists genetically alter nerve cells to produce light-sensing proteins, allowing them to control nerve activity with light. This could lead to advancements in the treatment of diseases like Parkinson’s and depression. The new device retains good performance after 3 months of soaking test and repeated pressing and friction for 5000 times. It is a useful multifunctional optogenetics tool and potentially integrated with wireless technology.

## Introduction

Considerable progress has been made in the last decade in optogenetics to manipulate specific circuits by excitation or inhibition of a specific neuron type with the expression of light-sensitive ion channels or ion pumps. This method is critically important in neuroscience research because of its capabilities in sophisticated functional studies in neural systems^1,2^. To deliver light to the targeted brain area, the conventional method is to insert an optical fiber into the brain tissue with an attachment at the opposite end to a remotely located laser or light-emitting diode (LED)^3,4^. However, the light sources have low spatial resolution due to the limited number of beams and the relatively imprecise positioning of the sources. To solve this problem, LED arrays^5^ or optical waveguide arrays^6,7^ have been applied. Additionally, because optical fibers can restrict the movement of the animal or even lead to entanglement, the ultra-miniaturized wireless-powered LED array is a superior choice^8,9^.

The subdural or epidural electrocorticography (ECoG) signals have been brought into focus in both research and application of functional and cognitive neuroscience due to the advantages of capturing broader band neural activity with higher spatial resolution^10^ and carrying more motor, cognitive, and language information for brain-computer interface control than electroencephalography^11^. Thus, ECoG is far more valuable and reliable with a high level of performance. To acquire ECoG signals, a high-density microelectrode array is usually utilized to record local field potentials from a large-area cortical surface at mesoscopic scales^12–14^. As the number of microelectrode sites increases, more information will be stored during multichannel recording. The entire size of the microelectrode array varies with the target cortical area of different animal species from mice^15^ and rats^16^ to primates^17^. However, few researchers have combined the micro-LED array with the microelectrode array for synchronous photostimulation and ECoG signal recording of nervous tissues^5,18^ in neural circuit research.

Advances in microelectromechanical system (MEMS) technologies have enabled precise fabrication of multichannel micro-LED arrays on flexible substrates^5,19^. Microelectrode arrays can also be obtained using similar MEMS fabrication approaches^5,13,20^. Compared to our previous work^21^, this paper has completely changed the device design by increasing the channel count from 3 micro-LEDs and 4 recording sites to 16 micro-LEDs and 16 recording sites. Now, this device is capable of simultaneous, high-resolution optical stimulation and electrical recording of a larger cortical area. In this work, we first describe the practical design for the application of the device on the cortical surface of a rat, as well as the detailed processes of the fabrication, including the respective flows of the micro-LED array and the microelectrode array. To ensure that the luminous surface of the micro-LEDs faces the same direction as the microelectrodes, wire-bonding technology is utilized for the electrical conduction instead of the low-melting-point solder bonding^5^ or the flip-chip bonding^22^. A key step in the wire-bonding connection is the silver paste-aided reinforcement that significantly improves wire-bonding strength and yield on the flexible polymer substrate. Accurate positioning of the micro-LEDs over the 4 × 4 holes on the microelectrode array is assisted by two temporary needles that penetrate through the identical alignment holes on the two sub-arrays into the poly-di-methyl-siloxane (PDMS) substrate.

For long-term implantation, the reliability of the device should be taken into consideration. Accordingly, we conducted a soaking test in the phosphate buffer solution (PBS) for 3 months and found little degradation in the electrical or optical performance. For the recording microelectrodes, a comparison of the electrochemical properties was performed before and after 5000 iterations of pressing or friction, and the performance declined very little as well. Moreover, in view of the potential thermal damage to the local tissue due to the heating of the micro-LED, the temperature increase of the micro-LED was studied specifically with different duty cycles (0.1–0.9), voltages (2.5–3.9 V), and frequencies (1–50 Hz), which can serve as a reference for safe optical stimulation. Finally, the flexibility and mechanical strength of two specimens with different thicknesses (5 and 10 μm) were evaluated by simulation and experiment, in which the thinner specimen (5 μm) is suitable for acute animal experiments due to better flexibility, whereas the thicker specimen (10 μm) is more durable and reliable in long-term implantation of free-moving rats.

## Materials and methods

### Design of the opto-electric neural interface

**Fig. 1:**
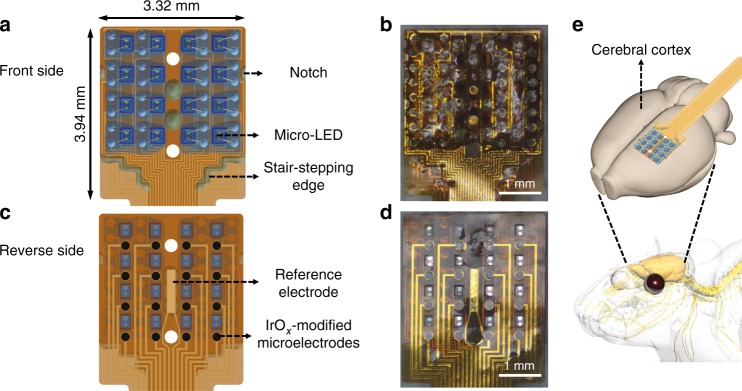
Design schematic of the opto-electric neural interface. **a** Schematic diagram and **b** photograph of the front side of the assembled device with 16 micro-LEDs on the top, **c** schematic diagram and **d** photograph of the reverse side of the assembled device with 16 IrO_*x*_-modified microelectrodes on the top, and **e** the sketch of the device attached on the cerebral cortex of a rat

The design objectives of this neural interface are opto-electric integration, mapping of the targeted brain area of a rat, and reliability for long-term use. The design schematic is shown in Fig. 1. This neural interface is composed of two individual parts with compatible fabrication processes, the 4 × 4 micro-LED array for optical stimulation, and the 4 × 4 microelectrode array for neural recording.

As illustrated in Fig. 1a, the overall size of the assembled device is 3.32 × 3.94 mm^2^, which matches the size of the cerebral hemisphere in rats. The photograph in Fig. 1b shows the front side of the fabricated device. In this assembly, the footprint of the micro-LED array is smaller than that of the microelectrode array with a stair-stepping end edge to apply the epoxy along this edge for adhesion of the two sub-arrays. Four circular holes are evenly distributed in the middle of the micro-LED array footprint, in which the two middle holes are used for dispensing the adhesive and the two side holes are used for the alignment with the same two holes on the microelectrode array. Additionally, two notches are symmetrically located at the edge of the footprint of the micro-LED array for dispensing the adhesive as well. To achieve relatively high resolution, according to the optimal spacing of the subdural, epidural, and scalp electrodes for rats as suggested by Slutzky and Kwon^23,24^, the pitch between the two neighboring micro-LEDs and for the two adjacent microelectrode sites is designed as 700 μm.

Sixteen (4 × 4) bare micro-LED chips (C460TR2227-0328, Cree Inc., USA), with dimensions of 220(*W*) × 270(*L*) × 50(*H*) μm^3^ and 460 nm in peak wavelength, are arranged in the array with the light-emitting surfaces facing downwards, allowing light to propagate through the holes without obstacles. Figure 1c, d shows the schematic diagram and photo of the reverse side view, where the 16-channel recording microelectrodes (200 μm in diameter) are distributed one-to-one next to the micro-LEDs and modified with iridium oxide (IrO_*x*_) for lower impedance and higher signal-to-noise ratio (SNR) during neural recording. Finally, a reference electrode is integrated in the middle of the microelectrode array.

All the anodes of the micro-LEDs are individually addressable, whereas their cathodes are connected together to reduce the number of interconnection leads. For optogenetics, the activation of the introduced light-sensitive ion channels and pumps, channelrhodopsin-2, requires an optical stimulus wavelength of 473 nm^25^, which is in the wavelength range of the micro-LED. The assembled device can be attached to the unilateral cerebral cortex of a rat as shown in Fig. 1e. The depth in which micro-LEDs evoke neural activity across different layers is expected to be over 600 μm as reported in the literature^5^. This design concept can be further expanded to larger or more complex areas, such as the cortical surface of nonhuman primates^26^ and the spinal cord and peripheral nervous system^27^, with more and smaller micro-LEDs and microelectrodes to realize more precise mapping, even utilizing color-tunable or multicolored micro-LEDs^28,29^ for simultaneous excitation and inhibition of neurons expressing different optogenetics opsins.

### Fabrication of the micro-LED array

**Fig. 2:**
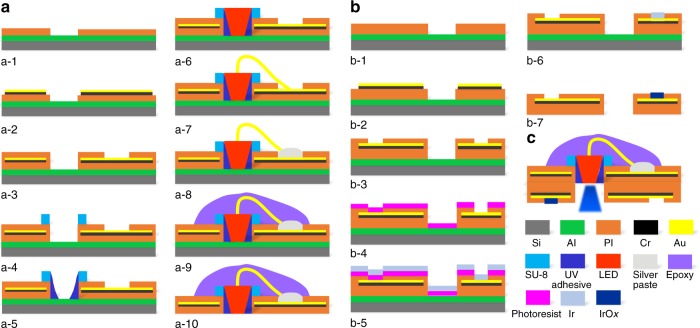
Fabrication processes. **a** The micro-LED array and **b** the micro-ECoG electrode array based on polyimide substrate. **c** Assembled device with the micro-LED array stacked on the micro-ECoG electrode array

**Fig. 3:**
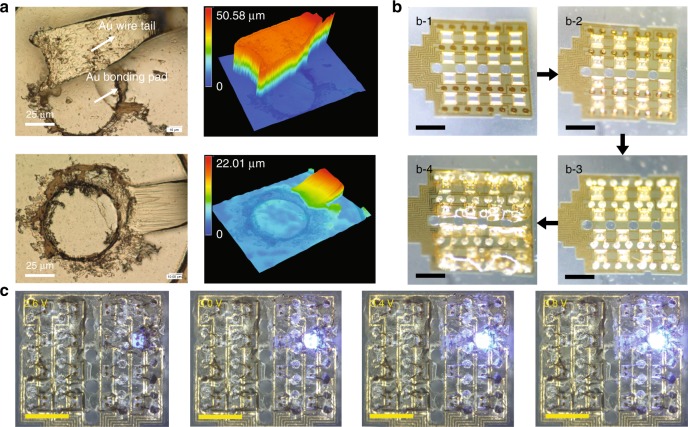
Silver paste-aided wire-bonding on the flexible substrate and its effect. **a** Two states of second bonding joint on polyimide substrate: disconnected (above) and connected (below). **b** Flow photos of the micro-LEDs’ setup and package showing the silver paste-aided wire-bonding. **c** Actual working effect of a single micro-LED under different voltages (2.6–3.8 V). All scale bars in **b** and **c** are 1 mm

**Fig. 4:**
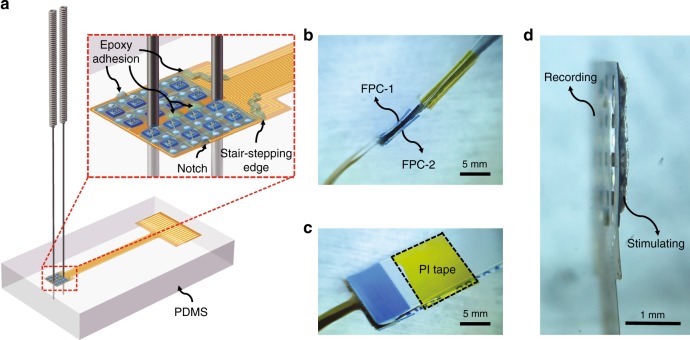
System assembly. **a** Schematic diagram of back-to-back alignment of two sub-arrays with two needles inserted through the identical round holes into a PDMS layer. The inset shows the two sub-arrays assembled together using epoxy dispensed at reserved positions. **b** Two flexible printed circuit boards (FPC-1 and FPC-2) hot-pressed to the contact pads of the micro-LED array and microelectrode array, respectively. **c** Bond of two FPCs close to the interfaces with a PI tape. **d** Side view of the assembled device with hybrid recording and stimulating capabilities

**Fig. 5:**
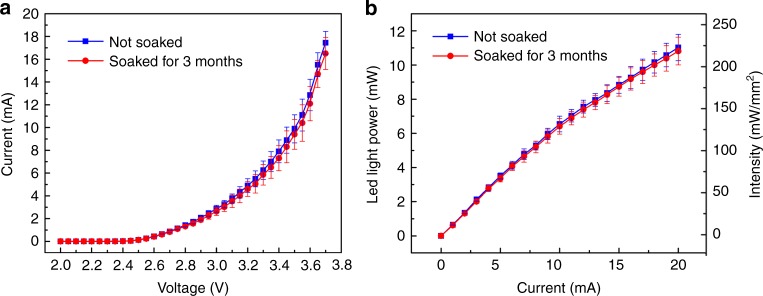
Electrical and optical properties of micro-LED arrays soaked in PBS for 3 months. **a** *I*–*V* mean value curves with standard deviations measured from 32 micro-LEDs on two samples. **b** Mean value and standard deviation of light output power and intensity of the same two samples

**Fig. 6:**
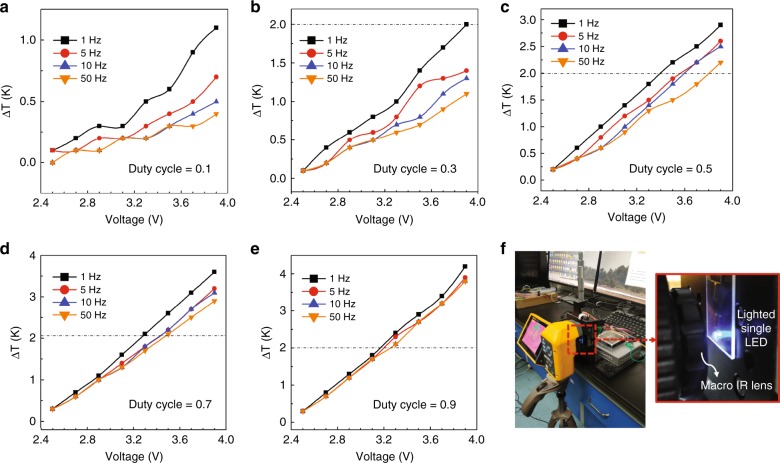
Thermal property of the integrated single micro-LED on the array. Duty cycle from 10–90% (**a**–**e**), frequency from 1 to 50 Hz, and voltage from 2.5 to 3.9 V; dashed line at ΔT = 2 K indicating the maximum acceptable temperature increase to avoid thermal damage of brain tissue. **f**Measurement setup with an IR thermal imager with the enlarged view of the micro-LED array facing the macro IR lens

**Fig. 7:**
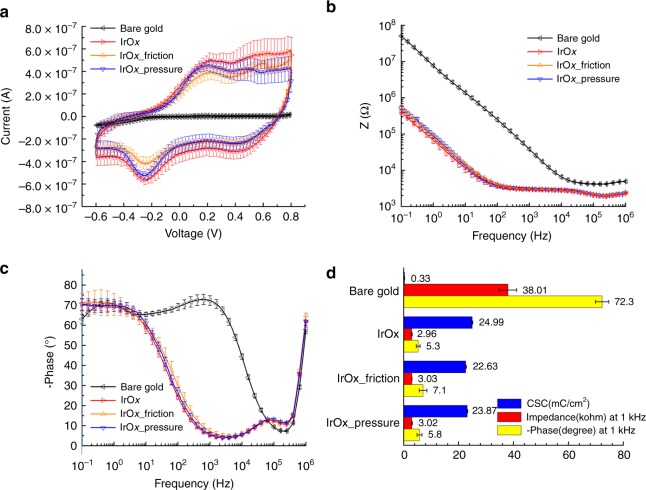
Electrochemical properties of IrO_*x*_-modified microelectrodes and their stability. **a** Cyclic voltammogram, **b** impedance, and **c** phase of two IrO_*x*_-modified microelectrode arrays (32 sites) before and after 5000 iterations of friction or pressing, compared with bare gold microelectrodes. **d** Comparison of bare gold and IrO_*x*_-modified microelectrodes before and after friction or pressing in charge storage capacity (CSC) as well as impedance and phase at 1 kHz

**Fig. 8:**
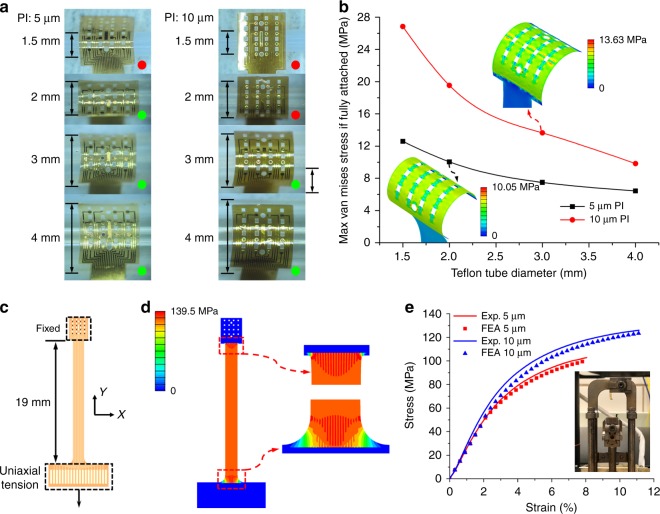
Flexibility and mechanical property of the microelectrode arrays with an overall thickness of 5 or 10 μm. **a** Attachment experiments of PI microelectrode arrays on Teflon tubes with different diameters of 1.5, 2, 3, and 4 mm. The red and green dots represent poor and good contact, respectively. **b** The simulated maximal stresses of the arrays with different thicknesses when fully attached to the Teflon tubes with the same diameters. The insets show the stress distribution of the fully attached 5 μm PI on a 2 mm tube and 10 μm PI on a 3 mm tube, respectively. **c** The diagram of the uniaxial tensile test of the microelectrode array with one side fixed and the other side under uniaxial tension. **d** Stress distribution of the PI microelectrode array with a thickness of 10 μm at the fracture critical state. **e** Experimental and FEA results of strain vs. stress curves of 5*-* and 10-μm-thick arrays. The inset shows the broken specimen in the DMA

The micro-LED array fabrication process can be subdivided into 10 steps, as reported in our previous work^30^. As shown in Fig. 2a, a-1, a 500-nm-thick aluminum (Al) is evaporated on a silicon wafer by physical vapor deposition serving as a sacrificial layer^31^, then the bottom layer of a photosensitive polyimide (PI, Durimide 7505, Fujifilm, Japan) is spun at speeds of 1500 and 3000 rpm on two separate wafers, followed by lithography and curing in N_2_ at 300 °C for 1 h to form approximately 5 and 2.5 μm in thickness, respectively. Cr/Au (20/300 nm) layers are deposited, covered by a patterned positive photoresist (3 μm), and etched using the ion-milling system (LKJ 150, Advanced Ion Beam Ltd, China) (Fig. 2a, a-2). The second PI layer is spun, patterned, and cured in N_2_ at 350 °C for 1 h to acquire another 5 or 2.5 μm thickness (Fig. 2a, a-3). Then, a 50 μm-thick SU-8 2025 (MicroChem Corp., USA) is spun on top, photoetched, and developed to form a fence structure with the inner dimension of 240 × 290 μm^2^ and width of 50 μm, just above the aligned rectangular holes on the PI layers (Fig. 2a, a-4). The adhesion force between the SU-8 and PI is sufficient for the following operations to proceed without the occurrence of detachment. This structure is used for more accurate and easier alignment and placement of the micro-LED chips, and the effect without and with the SU-8 fence is further discussed in Supplementary Figure S1. A moderate droplet of transparent ultraviolet (UV) curable adhesive is applied and gathered around the inwall of the SU-8 fence (Fig. 2a, a-5). The micro-LED chips are lifted and placed one by one into the SU-8 fence with the gold bonding pads facing upwards (Supplementary Figure S1) (Fig. 2a, a-6). The adhesive is cured under UV light to keep the micro-LED chips firmly attached to the SU-8 fence and the PI substrate. Wire-bonding is utilized connect the two gold bond pads (80 μm in diameter, as the first bonding joint) of the micro-LED and the corresponding gold pads (160 μm in diameter, as the second bonding joint) exposed on the top PI layer via the gold wire (25 μm in diameter) (Fig. 2a, a-7). Due to the poor adhesion of the second bonding joints and the gold pads on the flexible PI substrate, the conductive silver paste (H20E, Epoxy Technology Inc, USA) is dotted onto the second bonding joint and gold pads (Fig. 2a, a-8). After curing the silver paste at 80 °C for 2 h, the device is taken out and a moderate amount of transparent epoxy (PKM12C-1, Pattex, Germany) is applied to encapsulate the SU-8 fence, micro-LED and gold wires (Fig. 2a, a-9). Finally, the entire device is released from the wafer in the hydrochloric acid solution (Fig. 2a, a-10).

### Fabrication of the microelectrode array

The fabrication process for the microelectrode array shares the first three steps similar to the micro-LED array, as shown in Fig. 2, b-1 to b-3. Then, a layer of positive photoresist (30 μm) is spun and patterned to expose the 4 × 4 microelectrode sites (Fig. 2b, b-4). Ti/Ir (50/300 nm) layers are sputtered and patterned (Fig. 2b, b-5 and b-6) by lift-off in acetone to leave the microelectrode sites covered only by Ir. The array is released from the wafer in the hydrochloric acid solution and activated into iridium oxide, with a total thickness of 10 or 5 μm (Fig. 2b, b-7). There are three typical IrO_*x*_ fabrication methods: sputtering iridium oxide film^21^; activated iridium oxide film^32^; and electrodeposited iridium oxide film^33^. Considering the necessity of the most stable and lowest phase response in the neural recording, the activated iridium oxide is the best choice with a sweeping potential from −1.0 to +1.0 V at 0.05 Hz in PBS. After 300 activation cycles, the iridium is activated to iridium oxide. Finally, the two sub-arrays are glued together back to back by epoxy with the light of the micro-LEDs passing through the aligned openings, as illustrated in Fig. 2c.

### Silver paste-aided wire-bonding

According to Hall et al.^34^, soft polymers absorb ultrasonic energy and deform under the compressive loading of a rigid capillary, thereby reducing the wire-bonding strength and yield. In practice, it is highly difficult to detach the tail from the stitch on a PI substrate, and the tail easily separates from the gold bonding pad even with relatively proper bonding parameters (ultrasonic power, pressure, time, and temperature). Two states of a second bonding joint are shown using a digital microscope (VHX-5000, KEYENCE, Japan) in Fig. 3a, disconnected (above) and connected (below) with the same bonding parameters. It was expected that a few wires would be bonded, but it was unreliable due to the weak bonding force. Thus, the weak interface between the tail of the gold wire and the exposed gold film area is the main reason for failure during wire-bonding, and no exfoliation of Cr adhesion layer from the PI substrate is observed. To address this issue, the conductive silver paste is dispensed on the gold pads on the PI. The improved wire-bonding process is shown in Fig. 3b, including fabrication of the SU-8 fence array above the aligned rectangular holes (b-1), fixation of the micro-LED chips with UV curable adhesive in the SU-8 fences followed by wire-bonding (b-2), reinforcement by the conductive silver paste (b-3), and encapsulation by epoxy (b-4). As clearly seen in Supplementary Figure S2, although the bonding wires (approximately 270 μm in height) and epoxy encapsulation increase the total thickness (approximately 350 μm in height) from the top, they do not interfere with the recording microelectrodes on the reverse side when in use. After release, the silver paste-aided wire-bonding performs well for all micro-LEDs, which are tested one by one with a final yield of 95%. Three micro-LEDs failed on four samples in a total of 64 micro-LEDs due to the rupture of the exposed gold film or the connection of the micro-LED’s cathode and anode when the silver paste was inattentively dispensed. Here the actual working effect of a single micro-LED is presented in Fig. 3c under different voltages (2.6, 3.0, 3.4, and 3.8 V). The applied voltage upper limit is referred to the specifications of the micro-LED chip and the line resistance of the connection to the single micro-LED is approximately 40 Ω.

### Back-to-back alignment in assembly

To easily assemble the micro-LED array and microelectrode array together, a back-to-back manual assembly method is proposed, which is less dependent on costly instruments such as a high-precision flip-chip bonder. Here an elastic, flat PDMS piece is utilized as a platform to place and assist the assembly of the two sub-arrays. Figure 4a shows the schematic of the back-to-back alignment of the two sub-arrays with two steel needles inserted through the aligned round holes into the flat PDMS piece, in which the microelectrode array is beneath the micro-LED array with microelectrode sites facing the PDMS. The inset of Fig. 4a shows an enlarged view to show the epoxy distribution for adhesion, including the stair-stepping end edge, two identical round holes (300 μm in diameter) between the two needles, and two symmetrical notches at the edge on the top of the micro-LED array. These features are also shown in Figure S2c. The other ends of the two sub-arrays are separately hot-pressed to flexible printed circuits (FPC-1 and FPC-2) with the process described in Supplementary Figure S3. Then, the two FPCs are parallelly bonded together with the help of a thin PI tape (Fig. 4b, c). The side view of the final assembled device in Fig. 4d clearly shows both the recording and stimulating sides.

## Results and discussion

To verify the performance of the device, we studied the electrical, optical, and thermal properties of the micro-LED array, as well as the electrochemical, flexible, and mechanical properties of the microelectrode array. The results prove that this hybrid flexible neural system performs well and is promising for further animal experiments.

### Electrical and optical properties in a soaking test

The electrical and optical properties of the wire-bonded micro-LEDs should be quantified for better use of the device with proper stimulation parameters. In addition, the uniformity and long-term stability of the micro-LEDs on the array should be evaluated. Here we picked two samples with 32 micro-LEDs in total and tested every single micro-LED before and after soaking in PBS for 3 months at room temperature under wet conditions, during which all micro-LEDs were only soaked without operation. The *I*–*V* mean value curves with standard deviations were measured from the 32 micro-LEDs on the two samples by a semiconductor parameter analyzer (BA1500, Agilent Technologies, USA) as shown in Fig. 5a. The ratio of the standard deviation to mean value is <13% and 17% before and after soaking, respectively, and the current slightly decreases after soaking. This may be due to the slight resistance increase (<20 Ω) in the electrode leads and the silver paste. For the optical measurement, the light output was evaluated using a spectrometer (QE65 Pro, Ocean Optics) and an integrating sphere (FOIS-1, Ocean Optics), and the intensity was calculated by dividing the effective emission area of the micro-LED (190 × 240 μm^2^). As shown in Fig. 5b, the mean values and standard deviations of the overlapping light power and intensity curves are obtained from these two samples with the 32 micro-LEDs. Similarly, the ratio of the standard deviation to mean value is <7% and 8% before and after soaking, respectively, and the intensity shows a slight decrease after soaking as well. Considering the minimum light energy density of 1 mW/mm^2^ to induce neuronal action potentials^1^, the light intensity provided by our micro-LED could be sufficient for neurons in deeper layers. Our results indicate that the micro-LED array is uniform and reliable for long-term use in a liquid environment.

### Thermal property of the integrated micro-LED

Brain tissue damage caused by the heat production of the micro-LED is a major concern for in vivo applications. Accordingly, the temperature variation of a single micro-LED on the array is investigated using a thermal infrared (IR) imager (Fluke TiX 560, USA) with 0.05 K resolution and macro IR lens (Fluke Lens/25 Mac2, USA) with a pixel size as small as 25 microns. The test was performed in an enclosed environment with a constant room temperature at 20 °C where ventilation was strictly controlled. With the voltage increasing from 2.5 to 3.9 V and different frequencies ranging from 1 to 50 Hz, the rise in temperature of the single micro-LED was measured on the array floating in air with duty cycles from 0.1 to 0.9, corresponding to Fig. 6a–e. The dashed line indicates the maximal acceptable temperature increase (Δ*T* = 2 K) for brain implants^8^, which serves as a meaningful reference when conducting animal experiments. As the applied voltage increases, the temperature simultaneously increases. The difference in the temperature increase at varied frequencies is not obvious. However, the temperature increase distinctly rises with the duty cycle. The increase of duty cycle leads to an increased accumulation of heat in the micro-LED chip with less time to dissipate it. Therefore, the influence of the duty cycle is larger than that of the frequency. Figure 6f exhibits the measurement setup with an IR thermal imager, a micro-LED array fixed on a three-axis platform, and an LED power supply. The luminous surfaces of the micro-LEDs face the macro IR lens as shown in the enlarged view of Fig. 6f. One thing to note here is that the luminous surface of the micro-LED is not in direct contact with the brain tissue due to the clearance resulting from the thickness of the subjacent microelectrode array. Therefore, the temperature increase at the cortical surface should be lower, which implies that it is safe to apply a voltage higher than the maximal allowed value shown here.

### Electrochemical property of microelectrodes and stability

The microelectrode sites are modified with activated IrO_*x*_^32^ to improve the electrochemical performances at the electrode-tissue interface. Low microelectrode impedance is beneficial for acquiring high SNR in neural recording. Due to the likely friction or pressure-lead exfoliation of IrO_*x*_ during attachment and long-term use in animals, its stability is verified by comparing the cyclic voltammetry (CV), impedance, and phase in electrochemical measurements before and after 5000 iterations of friction or pressing (Fig. 7a–c). To elucidate the necessity of modification, bare gold microelectrodes (200 μm in diameter) are also tested as a comparison. Here four IrO_*x*_-modified microelectrode arrays and two bare gold arrays are chosen for the test. The IrO_*x*_-modified microelectrode arrays were measured before any operation, in which two of them underwent friction back and forth^21^, and the other two were under repeated compression^30^, both on a flat agar gel for 5000 iterations. The friction provides shear stress that is applied parallel to the surface of the microelectrode sites with no additional pressure from the top, which imitates the actual relative movement to the brain tissue. In addition, the nonnegligible swelling of brain tissue underneath the recording sites is also simulated by repeated lifting and pressing of a cotton swab on the microelectrode array with an average pressure of approximately 180 kPa. After, the arrays were measured again in PBS using a standard three electrodes system (PGSTAT12 Autolab, EcoChemie, Utrecht, Netherlands) with a saturated calomel electrode as the reference and a Pt sheet as the counter electrode. The mean value curves with standard deviations not only illustrate the uniformity of the 32 tested microelectrode sites and stability of IrO_*x*_ but also prove the great improvement in the electrochemical properties as a result of IrO_*x*_.

The charge storage capacity (CSC) is calculated from the CV in Fig. 7a, and values for the electrodes with bare gold, IrO_*x*_, IrO_*x*_ after friction, and IrO_*x*_ after pressing are compared in Fig. 7d and are 0.33 ± 0.05, 24.99 ± 0.22, 22.63 ± 0.27, and 23.17 ± 0.19 mC/cm^2^, respectively, in correspondence with previous results^32,35^. The CSC of the IrO_*x*_-coated electrode is approximately 76 times higher than that of the bare gold electrode and there are only a 10.4 and 7.3% decrease after friction and pressing. The impedance and phase at 1 kHz are presented in Fig. 7d. The impedance values are 38.01 ± 3.18, 2.96 ± 0.21, 3.03 ± 0.06, and 3.02 ± 0.16 kΩ and the phases are −72.3 ± 2.4°, −5.3 ± 0.7°, −7.1 ± 1.4°, and −5.8 ± 0.9° for the electrodes with bare gold, IrO_*x*_, IrO_*x*_ after friction, and IrO_*x*_ after pressure, respectively. These values are comparable to previous studies^32,36^. The impedance and phase of the IrO_*x*_-coated electrodes are less than one-tenth of bare gold, which are lowered by the cyclic activation and oxidation of Ir in PBS. In addition, no obvious change in the impedance or the phase is observed after friction and pressing.

### Flexibility of the microelectrode array

To study the flexibility of the two PI microelectrode arrays with thicknesses of 5 and 10 μm, the attachment experiment was conducted on cylindrical Teflon tubes with diameters of 1.5, 2, 3, and 4 mm as shown in Fig. 8a. These simplified cylinder models are small enough to be meaningful references to the cortex surface of a rat and easier to quantitatively study the flexibility. The red and green dots represent unwrapped and wrapped states, respectively. With only water capillarity as the adhesion force^37^, the thinner 5 μm PI achieves excellent conformal contact when the cylinder diameter is not <2 mm, whereas the thicker 10 μm PI can only wrap well on cylinders with diameters ≥3 mm.

To reveal the adhesion forces applied to the tubes, it was assumed that these two arrays could be fully attached on Teflon tubes and the maximum van Mises stresses were calculated in the finite element analysis (FEA) software ABAQUS (SIMULIA, Pawtucket, RI, USA) (Fig. 8b). The film devices were modeled by four-node composite shell elements (S4R) and the tubes were modeled by the hexahedron three-dimensional solid element (C3D8R). The surface traction was applied on the array area to realize complete contact with the tube. A displacement boundary restriction was applied to the middle line of the rectangle array area in the *x*-direction to keep the array facing the tube without rotation or misalignment. The insets of Fig. 8b show the van Mises stress distribution of the 5 μm PI when fully attached to a 2 mm tube and the 10 μm PI to a 3 mm tube corresponding to the minimum conformal wrapping curvatures observed in experiment. The maximum stresses are 10.05 and 13.63 MPa, respectively.

### Mechanical property of the microelectrode array

When conducting the animal experiment, the manual operation or stiffness mismatch between the flexible device and covered dental cement may induce a drawing force on the electrode. Therefore, it is necessary to evaluate the mechanical property to avoid tensile failure. Here two PI microelectrode arrays with thicknesses of 5 and 10 μm were tested. The sample on one side was fixed, whereas the other side was movable between the clamps in the dynamic thermomechanical analysis system (DMA Q800, TA Instrument, USA). The load was uniaxially applied from both sides at an initial length of 19 mm as shown in Fig. 8c. The ABAQUS enables studies of the mechanical response of the film devices under tension. They were modeled by shell elements with the entire array fixed in the upper dotted box and the uniaxial downward tensile force from the upper edge of the connection pad in the lower dotted box as shown in Fig. 8c. The 10-μm-thick specimen with the van Mises stress distribution when stretched to the critical fracture state is illustrated in Fig. 8d. The fracture first occurs on either end of the stripe due to the stress concentration in the connecting regions of the stripe and two ends, as shown in the enlarged views of Fig. 8d.

The experimental and FEA results of strain vs. stress are compared in Fig. 8e for both the 5 and 10 μm films. The experimental strain-stress curves show almost overlapping elastic deformation segments for the two specimens. Young’s modulus of *E* = 2.5 GPa can be calculated based on the experimental data. In the experiments, the cross-sectional area rapidly experienced necking in a localized region of the specimen when the loading was continued beyond the ultimate stress. The strains reached 8.0% and 11.1% for the 5 and 10 μm specimens, respectively, and the stresses reached 102.8 and 126 MPa when the fracture occurred, respectively. The thicker PI film has a larger elastic range than that of the thinner film. Additionally, the characteristics include elastic and plastic behaviors, but it is observably without the yield strength point due to the material properties. The FEA results match well with the experimental data from the elastic to necking state.

The tradeoff between mechanical strength and flexibility should be taken into consideration. As reported in a previous study^30^, the thinner sub-arrays can be independently attached on the cortical surface one by one without assembly in acute animal experiments, with all micro-LEDs precisely aligned to ensure that the light directly passes through the holes. However, to ensure adequate mechanical strength and avoid tensile failure in chronic implantation for freely moving animals, the thicker assembled sub-array is a better choice.

## Conclusions

In this paper, the design, fabrication, and characterization of an opto-electric interface combining 4 × 4 wire-bonded micro-LEDs and 4 × 4 IrO_*x*_-modified microelectrodes for simultaneous light stimulation and neural recording are presented. A key advantage of this novel design is that it enables the light of the micro-LED to directly pass through the holes without loss while using the microelectrode array to realize local mapping. With the use of the silver paste-aided wire-bonding method, the micro-LED array functions well. The uniformity of all micro-LEDs on the array and long-term stability after soaking also perform well. In addition, the thermal properties of the micro-LED on the array in this study is useful as a reference for light stimulation to avoid thermal damage to local tissue. The stability of activated IrO_*x*_-modified microelectrodes is validated by 5000 iterations of pressing or friction and the electrochemical performance declines very little. The 5-μm-thick microelectrode array achieves excellent conformal contact when the cylinder diameter is not <2 mm, whereas the 10-μm-thick array can only wrap well on cylinders with diameters ≥3 mm. For the mechanical strength of the 5- and 10-μm-thick devices, the stress reaches 102.8 and 126 MPa when fracture occurs, respectively. Owing to the widespread study of optogenetics in neural science and diseases, we believe this study will provide a useful, functionally integrated tool and insight to produce more precise opto-electric neural interfaces.

## Electronic supplementary material

Supplementary file
