# Supplementary material for: Flexible polyimide-based hybrid opto-electric neural interface with 16 channels of micro-LEDs and electrodes
Source: Microsyst Nanoeng. 2018 Oct 8;4:27. doi: 10.1038/s41378-018-0027-0 (PMC6220173; doi:10.1038/s41378-018-0027-0)
Supplement: Supplementary file 1 — Supplementary file [file 41378_2018_27_MOESM1_ESM.docx]

**Supplementary file**

**Advantages of the SU-8 fence structure.** At first, the fence structure was not applied. It led to the diffusion of UV adhesive out of control and difficulty in alignment of the micro-LED chip right to the target position. Then the SU-8 fence structures are added on the array, and it is much easier to put micro-LED chips inside with high accuracy and efficiency.

**Importance of a moderate droplet of UV adhesive.** If an excess droplet of UV curable adhesive is applied, the inner side of SU-8 fence will be filled like a pool, as shown in the left picture of Figure S1b. When the micro-LED chip is put inside, two main problems will arise. One problem is that the micro-LED chip would float on the adhesive, with downside partly or totally covered by UV adhesive. Besides, after curing, the yet soft adhesive will make it harder to utilize wire bonding on the micro-LED’s Au pad, due to the tilting and movement of the micro-LED chip under compressive loading of capillary when wire bonding. The other problem seems more serious when pressing the micro-LED chip down with the capillary. It will lead to the overflow of adhesive and may cover the Au pads on top surface of micro-LED. Then the capillary will be blocked by adhesive, and no gold ball will form at the tip of capillary during wire boding.

When a moderate droplet of adhesive is applied inside of the SU-8 fence, by a very fine stainless-steel wire contacting with the top edge of the fence, the UV adhesive will quickly move and disperse around the inwall, as shown in the right picture of Figure S1b. In this way, the downside surface of micro-LED chip will be exposed, and the adhesive can provide enough adhesion after UV light exposure.


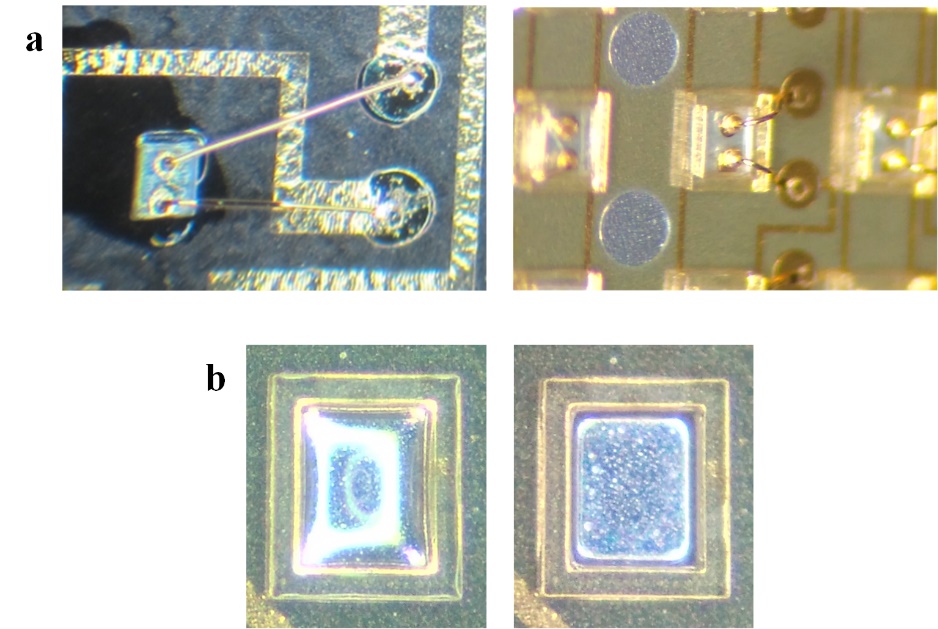


**Figure S1** (**a**) Comparison without (left) and with (right) the SU-8 fence structure. (**b**) Excess (left) and moderate (right) droplets of UV adhesive applied in the SU-8 fence.

**
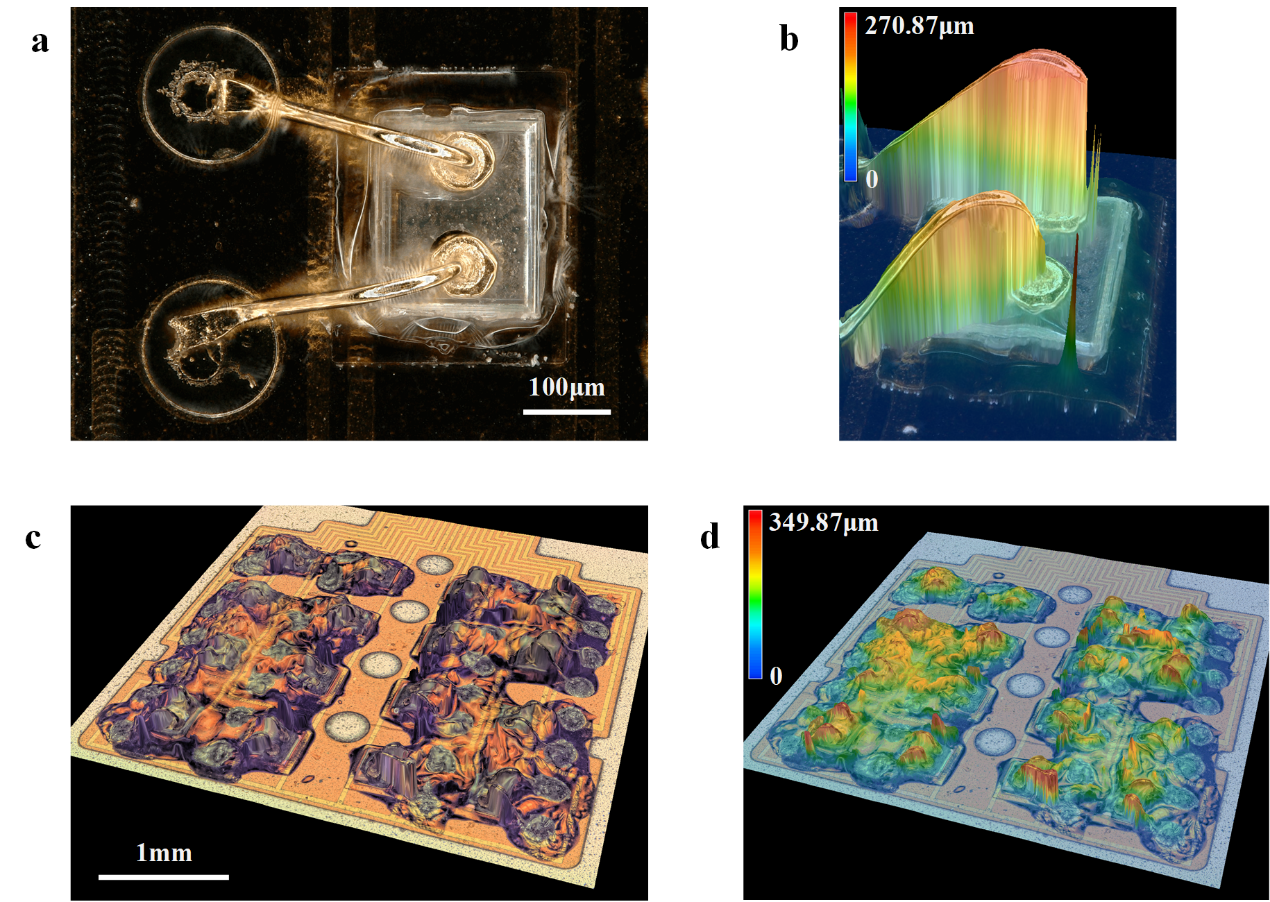
**

**Figure S2** Single wired micro-LED and the encapsulated micro-LED array pictured by the digital microscope. 3D image of the single wired micro-LED in (**a**) the picture model (top view) and (**b**) the height measurement model (isometric view). 3D image of the encapsulated micro-LED array in (**c**) the picture model and (**d**) the height measurement model.

**Hot-pressed process of the sub-array to FPC.** The two sub-arrays are individually connected to FPC with anisotropic conductive film (ACF, AC2056R, HITACHI, Japan) using a pulse hot-pressing machine (EN-600, Shenzhen Enormous Automaton Equipment Co., Ltd, China). With appropriate pressure (0.18 Mpa), temperature (240 ˚C), time (18 s) and hot-press direction (stiffener upwards), the connection between the electrode pad and FPC is reliable and durable.


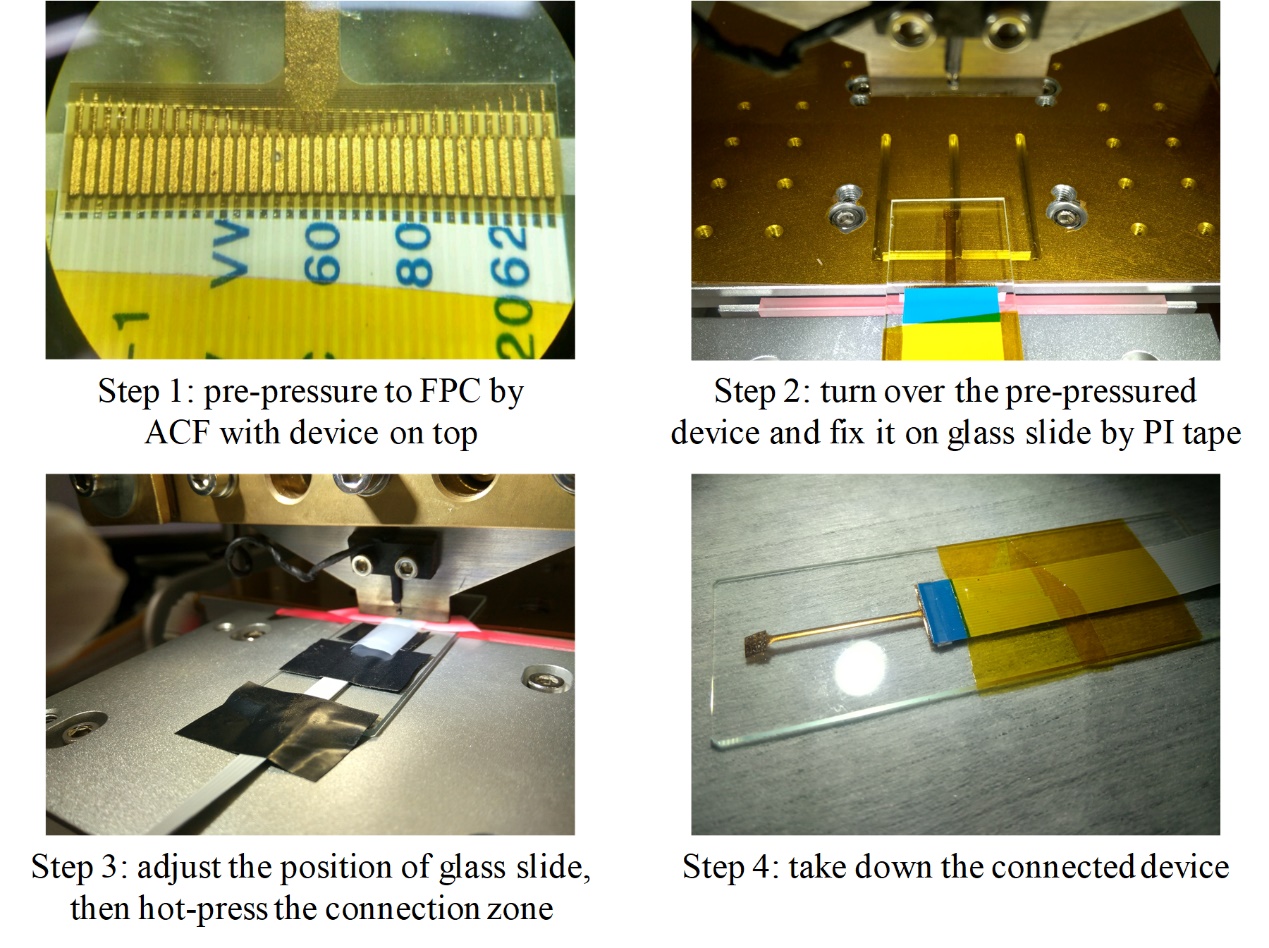


**Figure S3** Hot-pressed process of the sub-array to FPC.
